# Phytogenic zinc oxide nanoparticles in breast cancer therapy: an ethnopharmacological perspective on multi-targeted mechanistic pathways and translational constraints

**DOI:** 10.3389/fphar.2026.1883009

**Published:** 2026-10-09

**Authors:** Dhanya Soman Pillai, Ramesh Narve Venkatesha, Amarnath Karavettekudy Ranjit, Zeena Subramonia Pillai

**Affiliations:** 1 Department of Rasashastra and Bhaishajya Kalpana (Pharmaceuticals), Amrita School of Ayurveda, Amritapuri, Amrita Vishwa Vidyapeetham, Kollam, Kerala, India; 2 Department of Ophthalmology, Amrita School of Medicine, Amrita Vishwa Vidyapeetham, Kochi, India; 3 Department of Chemistry, Amrita School of Physical Sciences, Amritapuri, Amrita Vishwa Vidyapeetham, Kollam, Kerala, India

**Keywords:** 3D spheroids, apoptosis, breast cancer, green nanotechnology, phytogenic ZnO-NPs

## Abstract

**Introduction:**

Breast cancer remains the most frequently diagnosed malignancy and the leading cause of cancer death among women worldwide as of 2026. While conventional interventions face challenges like drug resistance and systemic toxicity, the integration of green nanotechnology with ethnopharmacology offers a biocompatible, sustainable alternative. This review assesses the mechanistic pathways and therapeutic potential of phytogenic zinc oxide nanoparticles (ZnO NPs) synthesized using bioactive secondary metabolites.

**Methods:**

This review combines findings from nine recent studies exploring biogenic ZnO NPs derived from diverse biological sources, including *Rhus coriaria* L. (Anacardiaceae), *Withania somnifera* (L.) Dunal (Solanaceae), *Zingiber officinale* Roscoe (Zingiberaceae). We critically analysed the characterization parameters, synthesis methods and pharmacological efficacy across 3D architectural spheroids, 2D monolayer cultures, and select *in vivo* models.

**Results:**

The collective data demonstrate that biogenic ZnO-NPs induce potent apoptosis through BAX upregulation, ROS-mediated mitochondrial dysfunction and specific cell cycle arrest. Notable benchmarks include tumour suppression comparable to Tamoxifen in *in vivo* models using melon-derived extracts and an IC50 of 14.7 μg/mL in 3D MCF-7 spheroids for ginger-mediated NPs.

**Discussion:**

In spite of the promising results, a critical methodological tension exists between limited preclinical validation and innovative synthesis. The pervasive reliance on 2D models often fails to duplicate the dense architectural barriers of solid tumours. Future research must prioritize a realistic translational pathway, shifting from basic *in vitro* models to rigorous *in vivo* biodistribution profiling, detailed metabolic tracking, and long-term toxicological assessments against normal mammary epithelial controls (e.g., MCF-10A) to actually establish, rather than merely hypothesize, a definitive, selective therapeutic index and ensure baseline physiological safety.

## 1 Introduction

Projections for 2026 point that breast oncology remains a cardinal global health challenge, marked by rising incidence rates and significant mortality among women. With an approximate 321,910 invasive cases expected in the United States alone during 2026, the global incidence continues to rise. Current clinical interventions, including chemotherapy and surgery, are often obstructed by the development of drug resistance and systemic toxicity. Accordingly, there is an essential call for effective risk reduction and alternative, targeted therapeutic strategies to overcome these clinical limitations and improve patient outcomes in modern oncology^1^ (Kaklamani and Arteaga, 2024; Wang and Wu, 2023).

Green nanotechnology integrated with ethnopharmacology offers a sustainable alternative to aggressive chemical synthesis. Extracts from *Cucumis melo* var. *inodorus* and *Ferula assa-foetida* facilitate the creation of biocompatible nanostructures, exploring metabolite synergies for improved molecular targeting (Łukowiak and Stolarczyk, 2026). Similarly, zinc oxide nanoparticles synthesized using *Santalum album* represent a frontier in breast cancer therapy (Alqahtani et al., 2025). By aligning historical Ayurvedic and traditional Chinese medicine rationales with modern green synthesis, researchers are repurposing traditional attributes to drive apoptotic signalling (Mahendra and Bisht, 2012). These biogenic platforms serve as adaptable, low-cost models for future drug delivery and high-potency, multi-targeted therapies (Guo and Morshedi, 2025).

Critically, the successful clinical translation of these nanomedicines demands a shift from simplified 2D monolayer cultures to 3D architectural spheroids that accurately duplicate dense tissue barriers. Moreover, establishing comparative benchmarks against gold-standard treatments, is essential to quantify relative therapeutic efficacy. Without rigorous *in vivo* validation, clinical trials and pharmacokinetic profiling, the systemic stability and long-term safety of these plant-capped structures remain theoretical (Guo and Morshedi, 2025).

It is against this translational backdrop that this review establishes its independent novelty relative to broad, cross-malignancy surveys such as the 2024 review by Rodríguez-Barajas et al. (2024). While prior literature provides a generalized compilation across multiple cancer types, this review establishes a distinct, specialized positioning within the ethnopharmacological scope. It achieves this by executing a targeted methodological critique of 2D versus 3D architectural spheroids, isolating plant-capped formulations benchmarked against clinical gold standards like Tamoxifen, and systematically tracking modern molecular mechanisms such as BAX upregulation, S-phase arrest, and microRNA modulation.

## 2 Methodology

### 2.1 Literature search methodology

To ensure transparency and a systematic approach to this narrative mini-review, a structured literature search was executed to identify key studies evaluating the efficacy of phytogenic zinc oxide nanoparticles (ZnO NPs) specifically in breast cancer models. This manuscript is structured as a focused narrative mini-review prioritizing a detailed multi-targeted mechanistic and architectural critique of nine distinct biogenic sources.

#### 2.1.1 Search databases and timeline

Comprehensive electronic searches were conducted across PubMed/MEDLINE, Scopus and Google Scholar. The literature search was initially performed in January 2026, with an updated literature sweep finalized up to May 2026 to capture the most recent advancements.

#### 2.1.2 Search strategy

Keyword combinations and Boolean operators utilized during the search included, (“zinc oxide nanoparticles” OR “ZnO NPs” OR “biogenic” OR “green synthesis” OR “phytogenic”) AND (“breast cancer” OR “MCF-7” OR “MDA-MB-231”) AND (“ethnopharmacology” OR “plant extract” OR “phytochemical”).

#### 2.1.3 Inclusion criteria

Primary peer-reviewed research articles evaluating green-synthesized or biogenic ZnO NPs using characterized biological extracts. Studies featuring clear *in vitro* (2D or 3D spheroids) or *in vivo* breast cancer therapeutic evaluation. Articles containing distinct data on characterization, dose or IC_50_, or exact molecular or cellular mechanisms of action.

#### 2.1.4 Exclusion criteria

Dissertations, book chapters, conference abstracts, studies focusing exclusively on purely chemical or physical nanoparticle synthesis, and studies detailing anti-cancer efficacy on non-breast malignancies without breast cancer comparative data. Applying these stringent selection criteria yielded the core cohort of nine structurally distinct studies detailed comprehensively throughout this review (Table 1; Figure 1).

**TABLE 1:** Per-study extraction: biogenic synthesis and anticancer evaluation of ZnO-NPs.

Material (phytochemical source)	Model (cell line/Organism)	Dose/IC50	Main outcomes	Mechanisms of action	Limitations
*Zingiber officinale* Roscoe (Zingiberaceae) (Alhaddad et al., 2024)	3D MCF-7 spheroids	IC50: 14.7 μg/mL	Complete spheroid destruction; bypasses 2D limitations	TNF-α upregulation; inflammatory-mediated cytotoxicity	Large particle size (171–195 nm); lacks non-malignant safety profiles
*Rhus coriaria* L. (Anacardiaceae) (Mongy and Shalaby, 2024)	MDA-MB-231 (TNBC)	IC50: 55.54–63.71 μg/mL	81.57% apoptotic rate; suppressed metastatic potential	S-phase cell cycle arrest; biogenic hexagonal wurtzite integration	Lack of *in vivo* data and comparative toxicity against normal cells
*Cucumis melo* var. inodorus Jacq. (Cucurbitaceae) (Mahdizadeh et al., 2019)	MCF-7, TUBO (murine); *in vivo* tumor model	Dose-dependent cytotoxicity	Tumor suppression comparable to Tamoxifen	Extrinsic apoptotic pathway; Caspase-3 and Caspase-8 upregulation	Limited data on long-term metabolic clearance and pharmacokinetics
*Ferula assa-foetida* L. (Apiaceae) (Boskabadi et al., 2020)	MCF-7, MDA-MB-231, HT-29	Dose-dependent cytotoxicity	Potent action across breast and colon cancer cell lines; selective over HUVEC.	BAX gene upregulation; Sub-G1 phase arrest	Reliance on 2D monolayers; antioxidant claims need reconciliation with ROS.
*Withania somnifera* (L.) Dunal (Solanaceae) (Prasad et al., 2021)	MCF-7	Dose-dependent cytotoxicity	Robust programmed cell death via bioactive metabolites	Inferred ROS overproduction; mitochondrial dysfunction	Lacks structural complexity (3D) and *in vivo* efficacy benchmarks
*Santalum album* L. (Santalaceae) - biogenic nanorods (Kavithaa et al., 2016)	MCF-7	Inhibits proliferation	Effective inhibition of cell proliferation	Intrinsic apoptotic signaling; Sub-G1 phase arrest via oxidative stress	Neglects extrinsic mechanisms; lacks 3D architectural penetration data
*Albizia lebbeck* (L.) Benth. (Fabaceae) (Umar et al., 2019)	MCF-7, MDA-MB-231	Dose-dependent cytotoxicity	66 nm nanoparticles; synergistic antitumor, antioxidant, and antimicrobial activity	Phytochemical synergy from stem bark-derived agents	Absence of *in vivo* validation and 3D tumor barrier testing
*Vateria indica* L. (Dipterocarpaceae) (D’Souza et al., 2021)	MDA-MB-468 (TNBC)	Dose-dependent cytotoxicity	Inhibition of proliferation	Intracellular ROS induction; apoptotic signaling	Reliance on 2D models; lacks precise cell cycle (S-phase) data
*Saponaria officinalis* L. (Caryophyllaceae) (Aalami et al., 2020)	Breast cancer cells	Dose-dependent cytotoxicity	Epigenetic reprogramming and potent antiproliferative effects	Modulation of microRNAs (miRNAs); apoptotic induction	Lacks *in vivo* benchmarks (Tamoxifen) and 3D spheroid validation

**FIGURE 1:**
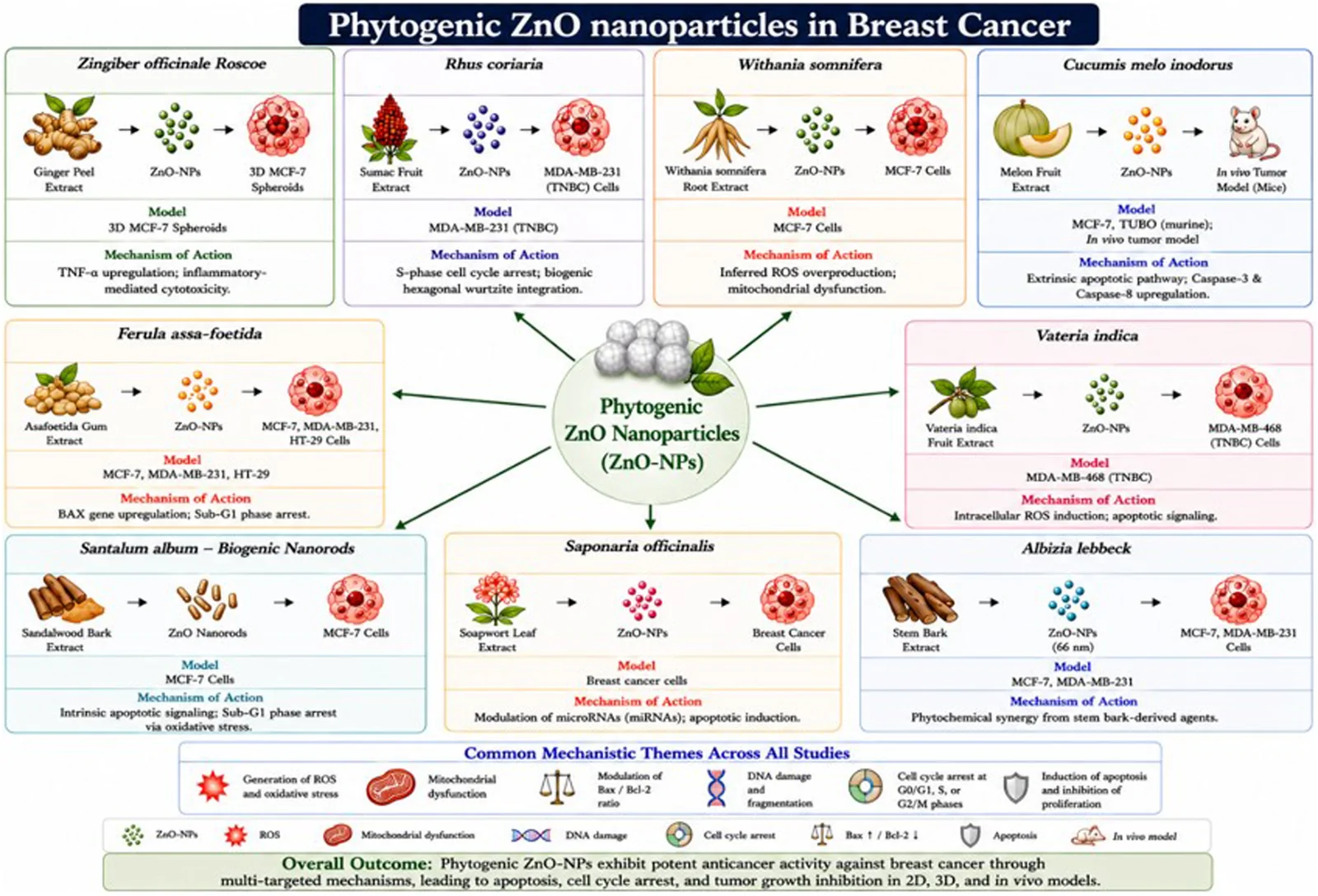
Anticancerous activity of Green synthesised ZnO nanoparticles.

### 2.2 Preparation of green-synthesized ZnO nanoparticles

The green synthesis of ZnO NPs (Figure 2; Table 2) represents a sustainable ethnopharmacological approach, using bioactive secondary metabolites as reducing and stabilizing agents to eliminate toxic reagents. Aqueous extracts from sources (Farooqi et al., 2026; Habtemariam et al., 2026), such as *Albizia lebbeck* (L.) Benth. (Fabaceae) stem bark and *C. melo* var. *inodorus* Jacq. (Cucurbitaceae) fruit, are combined with zinc precursors via eco-friendly sol-gel or biomimetic methods. Metabolites like gingerols act as capping agents, influencing morphologies from hexagonal wurtzite structures to complex flower-like geometries. Additionally, polysaccharides from brown seaweed (*Sargassum muticum*) (Namvar et al., 2016) and marine microbial metabolites (*Bacillus cereus*) (Ahmed et al., 2021) provide alternative pathways for synthesizing biocompatible nanostructures. The quality and pharmacological relevance of the included natural products were assessed according to the GP-TCM RA best practice guidelines provided in the Supplementary File 1

**FIGURE 2:**
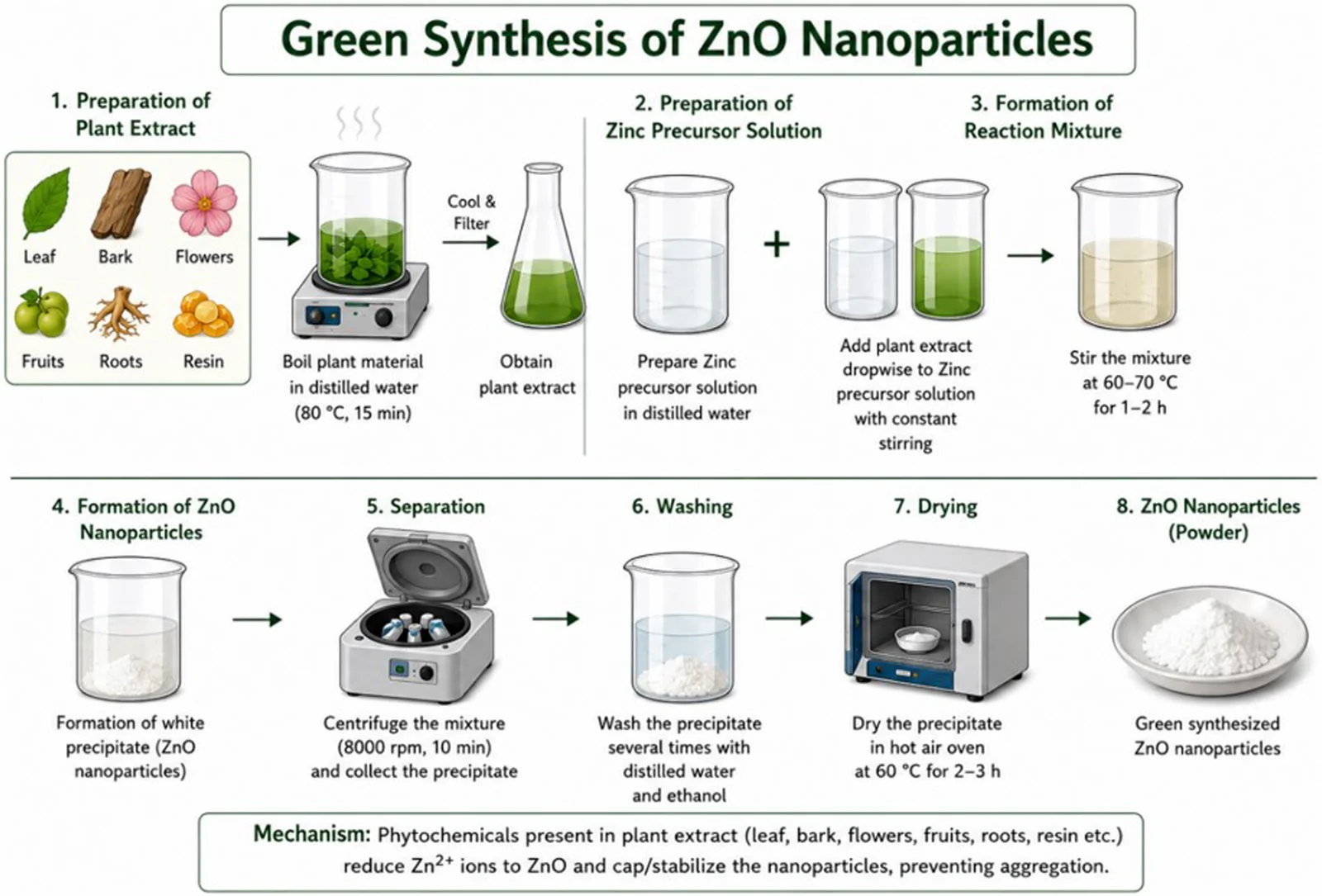
Preparation of phytogenic zinc oxide nanoparticles.

**TABLE 2:** Comparative synthesis, characterization and pharmacological efficacy of phytogenic ZnO nanoparticles.

ZnO nanoparticle type	Taxonomic identification (Family)	Biological source	Brief Preparatory method	Characterization (Size/Morphology)	Reference
Ginger-mediated	*Zingiber officinale* Roscoe (Zingiberaceae)	Rhizome extract	Green synthesis using gingerol-rich extracts	171–195 nm; enhanced 3D penetration	Alhaddad et al. (2024)
Sumac-mediated	*Rhus coriaria* L. (Anacardiaceae)	Fruit extract	Biogenic synthesis with sumac metabolites	∼20 nm; Hexagonal wurtzite	Mongy and Shalaby (2024)
Melon-mediated	*Cucumis melo* var. *inodorus* Jacq. (Cucurbitaceae)	Fruit extract	Green synthesis for targeted delivery	Plant-capped structures	Mahdizadeh et al. (2019)
Assafoetida-mediated	*Ferula assa-foetida* L. (Apiaceae)	Resin/Extract	Biogenic fabrication using resin metabolites	40 nm structures	Boskabadi et al. (2020)
Ashwagandha-mediated	*Withania somnifera* (L.) Dunal (Solanaceae)	Root extract	Synthesis utilizing bioactive withanolides	Spherical biogenic structures	Prasad et al. (2021)
Biogenic Nanorods	*Santalum album* L. (Santalaceae)	Biological extract	Facile biosynthesis of one-dimensional rods	Rod-shaped morphology	Kavithaa et al. (2016)
Siris-mediated	*Albizia lebbeck* (L.) Benth. (Fabaceae)	Stem bark	Biosynthesis from bark-derived phytochemicals	66 nm nanoparticles	Umar et al. (2019)
White Dammar-mediated	*Vateria indica* L. (Dipterocarpaceae)	Fruit metabolites	Biocompatible nanostructure fabrication	Biogenic nanostructures	D’Souza et al. (2021)
Soapwort-mediated	*Saponaria officinalis* L. (Caryophyllaceae)	Leaf extract	Synthesis targeting breast cancer cells	Capped nanostructures	Aalami et al. (2020)

### 2.3 Toxicological profile and safety parameters of ZnO nanoparticles

ZnO NP toxicity involves mitochondrial dysfunction, oxidative stress, and DNA damage (Liu et al., 2017). Safety profiles vary by exposure route; sub-chronic dermal applications in rats show minimal systemic effects, with a NOAEL of 1,000 mg/kg (Ryu et al., 2014; Dorato and Engelhardt, 2005). Conversely, inhalation studies reveal pulmonary inflammation and metal ion release, identifying the respiratory system as a sensitive target (Adamcakova-Dodd et al., 2014). While the oral LD50 in rodents exceeds 2,000 mg/kg (Walum, 1998), aquatic models like Javanese medaka exhibit higher sensitivity (Amin et al., 2021). Overall, while bulk ZnO is generally safe, nano-counterparts require strict dosage tracking to mitigate cellular toxicity (Vandebriel and De Jong, 2012).

### 2.4 Anticancerous activity of green-synthesized ZnO nanoparticles

#### 2.4.1 Oxidative stress induction, mitochondrial dysfunction, and intrinsic apoptosis

Biogenic zinc oxide nanoparticles (ZnO-NPs) exploit semiconductor redox capacity to induce intracellular reactive oxygen species (ROS), triggering structural damage. Prasad et al. (2021) utilized *Withania somnifera* (L.) Dunal (Saleem et al., 2020) (Ashwagandha) to synthesize structures exhibiting dose-dependent cytotoxicity against MCF-7 cells. This efficacy, driven by mitochondrial dysfunction and caspase-mediated apoptosis, validates Ashwagandha’s traditional status as a *Rasayana* (rejuvenator). Ultimately, nano-scale oxidative stress bridges ancient medical claims regarding this root’s ability to curb malignant growth by balancing cellular energy.

However, a notable translational limitation remains; this evaluation depends almost exclusively on simplified *in vitro* assays, leaving the crucial long-term biocompatibility, systemic biodistribution, and metabolic clearance of these biogenic nanoparticles within complex *in vivo* mammalian models completely unresolved. Moreover, the specific metabolites responsible for the reduction and stabilization of the ZnO core remain only qualitatively identified, necessitating future rigorous bio-guided fractionation to properly standardize the therapeutic efficacy required for standardized clinical ethnopharmacological applications.

Kavithaa et al. (2016) engineered *Santalum album* L. nanorods to target MCF-7 cells (Kavithaa et al., 2016; Kumar et al., 2015). These structures induce dose-dependent cytotoxicity by triggering the intrinsic apoptotic pathway through oxidative stress. This mechanism mirrors the traditional anti-inflammatory profile of sandal wood, confirming its potential as a precise inhibitor of aberrant cellular proliferation.

Nevertheless, this platform faces major limitations: the study focuses exclusively on intrinsic signalling mechanisms, completely neglecting the dual caspase-3 and caspase-8 extrinsic cross-talk networks established in contemporary dual-model oncological evaluations. Furthermore, the complete reliance on flat 2D monolayer cultures fails to account for the dense architectural and interstitial fluid barriers, characteristic of real solid tumours, and without comparative *in vivo* benchmarks against gold-standard clinical chemotherapeutics, the translation viability of these sandalwood-capped nanorods remains strictly preliminary.

Expanding this redox paradigm, D’Souza et al. (2021) investigated *Vateria indica* L. fruit-derived matrices to synthesize biocompatible ZnO nanostructures targeting triple-negative breast cancer (TNBC) (D’Souza et al., 2021; Isaq et al., 2022). These structures display optimized dose-dependent cytotoxicity against MDA-MB-468 cells (D’Souza et al., 2021; Wojtowicz et al., 2020). The mechanism involves intracellular ROS induction, triggering apoptotic signalling to arrest malignant proliferation. This targeted suppression of challenging TNBC phenotypes honours the traditional ethnopharmacological heritage of White Dammar, whose resin is used for treating severe ulcers and hard swellings. This approach successfully bridges historical tissue-remodelling practices with the modern oncological necessity of targeting metastatic breast cancer.

Despite these promising outcomes, the clinical transition of these nanostructures is impeded by profound challenges regarding long-term systemic toxicity and the metabolic clearance rates of the plant-capped cores within complex biological systems. Furthermore, the inherent seasonal and geographic variability in metabolite yields from *V. indica* L. (Dipterocarpaceae) fruits requires strict standardization of the biogenic synthesis process to ensure batch-to-batch pharmacological consistency. Future investigations must prioritize complex *in vivo* pharmacokinetic modelling to validate the systemic bioavailability and targeted delivery kinetics of these nanostructures within the tumour microenvironment.

#### 2.4.2 Receptor-mediated extrinsic apoptotic cascades and *in vivo* efficacy

To achieve therapeutic parity, researchers are targeting death receptor-mediated mechanisms. Mahdizadeh et al. (2019) synthesized *Cucumis melo* var. *inodorus* Jacq.-capped ZnO nanoparticles, demonstrating dose-dependent cytotoxicity in human MCF-7 and murine TUBO lines (Mahdizadeh et al., 2019; Kapoor et al., 2020; Zhang et al., 2025). These particles trigger programmed cell death via Caspase-3 and Caspase-8 upregulation (Pu et al., 2016). Systemic administration achieved tumour suppression comparable to Tamoxifen, validating traditional reputation of *C. melo* var. *inodorus* Jacq for treating swellings and bridging ancient clinical intuition with modern oncological benchmarks.

However, a critical gap in this study is the limited data provided regarding the long-term pharmacokinetic behaviour and metabolic clearance of these plant-capped structures. A more exhaustive, multi-organ evaluation of chronic systemic toxicity, alongside mandatory *in vitro* screening against non-malignant mammary epithelial cell lines like MCF-10A, is required to define a precise selective therapeutic index before clinical translation can be safely contemplated.

#### 2.4.3 Genetic upregulation of BAX and sub-G1 phase cell cycle arrest

Altering the ratio of pro- and anti-apoptotic proteins while locking the cell cycle provides a potent mechanism of action. Boskabadi et al. (2020) synthesized 40 nm biogenic zinc oxide nanoparticles using *Ferula assa-foetida* L. resin metabolites, demonstrating dose-dependent cytotoxicity across MCF-7, MDA-MB-231, and HT-29 cancer models (Boskabadi et al., 2020; Amalraj and Gopi, 2016). Upregulation of the pro-apoptotic BAX gene triggers mitochondrial permeabilization and programmed cell death (Wang et al., 2024). Additionally, these nanoparticles induce Sub-G1 cell cycle arrest while maintaining selectivity over non-cancerous HUVEC cells (Peng et al., 2023; Shioda et al., 2018). This confirms that traditional medicinal uses for dissolving internal masses translate directly into effective, targeted molecular apoptosis. Despite these apoptotic improvements, the current research depends heavily on simplified 2D monolayer cultures that fail to replicate the dense, multi-layered architectural barriers of solid tumours. Moreover, the claims of the author regarding secondary antioxidant activity require more rigorous alignment with the oxidative stress typically required for ZnO-mediated therapeutic efficacy.

This apparent paradox is resolved by a concentration-dependent biphasic switch. The antioxidant metabolites present within the *F. assa-foetida* oleo-gum-resin cap act as protective radical scavengers at low systemic concentrations, protecting healthy lineages. However, once localized within the tumour environment, the high structural density of the plant-capped nanostructures delivers a concentrated multi-targeted attack where the physical breakdown of the ZnO core forces heavy mitochondrial collapse, rendering the secondary antioxidant matrix incapable of neutralizing the localized, dominant pro-oxidant cascade.

#### 2.4.4 S-phase cell cycle arrest and anti-metastatic activity in TNBC

Targeting DNA replication is crucial for preventing the proliferation of highly metastatic breast cancer. Mongy and Shalaby (2024) authenticated *Rhus coriaria* L.-mediated ZnO-NPs against triple-negative breast cancer cell lines (Mongy and Shalaby, 2024; Alsamri et al., 2021). This synthesis yields crystalline hexagonal wurtzite structures, facilitating enhanced biological integration. These nanoparticles exhibit dose-dependent cytotoxicity in MDA-MB-231 cells (Snedec et al., 2025), identifying a therapeutic window that drives an 81.57% apoptotic rate and specific S-phase cell cycle arrest (Laskey et al., 1989). This suppression aligns with the traditional use of Sumac as an astringent and tissue-fortifying agent, providing molecular validation for how its therapeutic profile arrests aggressive cellular transformations.

However, the total dependency of this study on *in vitro* models limits our understanding of how these wurtzite particles behave in complex systemic environments. Moreover, the complete absence of comparative toxicity data against non-cancerous cell lines, such as MCF-10A, makes it impossible to evaluate the selective toxicity and overall safety profile of these nanoparticles for clinical translation.

#### 2.4.5 Epigenetic reprogramming via microRNA modulation

Aalami et al. (2020) utilized *Saponaria officinalis* L. leaf extract to synthesize ZnO nanoparticles for breast cancer therapy (Aalami et al., 2020; Chandra and Rawat, 2015). These nanostructures demonstrate potent antiproliferative effects by modulating microRNAs alongside traditional apoptosis (Aalami et al., 2020). This epigenetic reprogramming increases selectivity against malignant lineages, providing a molecular basis for traditional depurative use of Soapwort and demonstrating that these remedies actively alter cellular behaviour at a foundational level.

However, a major preclinical limitation remains the potential for off-target miRNA modulation in surrounding healthy tissues, which poses a serious risk of unintended systemic epigenetic alterations. Furthermore, the precise molecular mechanisms governing how the *S. officinalis* capping agents integrate with the ZnO core to selectively alter specific miRNA expressions require deeper transcriptomic and next-generation sequencing authentication. Translating these *in vitro* epigenetic insights into clinical applications is further hindered by a complete lack of *in vivo* stability and delivery kinetics data for these biogenic nanoparticles.

#### 2.4.6 Inflammatory-mediated 3D spheroid disruption and tumour architecture deconstruction

Bypassing the limitations of conventional monolayer testing is crucial for understanding how nanoparticles penetrate physical tumour masses. Alhaddad et al. (2024) advanced ethnopharmacological nanotechnology by synthesizing ZnO NPs using *Zingiber officinale* Roscoe (Zingiberaceae) (Alqahtani et al., 2025; Mao et al., 2019) rhizome extract against breast cancer (Alhaddad et al., 2024), employing gingerol-rich extracts traditionally utilized for their potent anti-inflammatory properties. By utilizing complex 3D MCF-7 spheroids (Chen et al., 2022) to entirely avoid 2D monolayer limitations, the study reported a highly effective observed IC50 of 14.7 μg/mL for its 2% formulation.

This efficacy, causing spheroid disintegration, is attributed to TNF-α upregulation (Jang et al., 2021). Although these biogenic structures (171–195 nm) exceed 100 nm, their enhanced penetration relies on biochemical deconstruction. A surface corona of [6]-gingerol (Gawel et al., 2021) disrupts tissue junctions, facilitating deep entry into the core. However, this size challenges long-term systemic clearance. Furthermore, the absence of non-malignant epithelial controls limits defining a selective therapeutic index. These methodological gaps, including reliance on simplified models and missing pharmacokinetic data, require comprehensive addressal in future research.

This potent destruction of dense tumour architecture directly mirrors the traditional ethnomedical deployment of *Z. officinale* Roscoe (Zingiberaceae) in Ayurvedic and indigenous systems to clear deep-seated inflammatory pathologies, hard tissue indurations, and chronic internal obstructions. By transitioning these traditional anti-inflammatory and tissue-penetrating attributes into green nanotechnology, modern nanomedicine validates the historical use of ginger rhizomes in resolving abnormal growth masses.

#### 2.4.7 Multi-targeted phytochemical synergy and broad-spectrum efficacy

Certain biogenic platforms achieve efficacy through multi-targeted biochemical synergy. Umar et al. (2019) evaluated the biosynthesis of 66 nm zinc oxide nanoparticles using *A. lebbeck* (L.) Benth. stem bark extract as a multi-targeted ethnopharmacological approach (Umar et al., 2019; Balkrishna et al., 2022). These nanostructures exploit bark-derived metabolites to smoothen biological integration, illustrating potent dose-dependent cytotoxicity against both MDA-MB-231 and MCF-7 breast cancer cell lines (Umar et al., 2019). The pharmacological profile is characterized by a synergy of antimicrobial, antioxidant, and antitumor activities. This multifaceted action successfully mimics the traditional Ayurvedic indications of *Albizia lebbeck* stem bark, confirming that its complex phytochemicals can be harnessed to disrupt multi-lineage cancer cell lines.

Nevertheless, this investigation lacks the mechanistically distinct S-phase arrest or high-potency 3D spheroid destruction observed in more advanced biogenic evaluations. Furthermore, the lack of living system validation and total dependence on flat monolayer models remains a major obstacle to confirming if these bark-derived structures can bypass physiological barriers. Without establishing comparative benchmarks against gold-standard oncology drugs, the systemic viability of this platform remains largely theoretical.

#### 2.4.8 Ethnopharmacological relevance, traditional clinical rationale, and ancient nanotechnology foundations

Traditional Indian Ayurvedic pharmacy (Rasashastra) pioneered the use of nanoparticles through a sophisticated, cyclical pharmaceutical process known as *Marana*. This process transforms bulk metals and minerals into *Bhasmas*—biocompatible, herbometallic nanoparticles characterized by their unique therapeutic properties and high bioavailability (Chandran et al., 2019). Yashada Bhasma, a direct traditional counterpart to green-synthesized ZnO-NPs, is valued for its tissue-rejuvenating and anti-inflammatory properties. Modern green nanotechnology evolves these principles, using bioactive metabolites as capping agents to mirror traditional detoxifying workflows. Botanical agents carry documented clinical rationales: *W. somnifera* (Ashwagandha) (Singh et al., 2011) is a traditional rejuvenator, mirroring modern anticancer efficacy via ROS generation and mitochondrial collapse; *Z. officinale* (Ginger) (Dissanayake et al., 2020) is used to clear obstructions, now shown to physically deconstruct dense 3D tumour architectures; *F. assa-foetida* (Mahendra and Bisht, 2012) and *S. album* (Choudhary and Chaudhary, 2021) treat internal masses, authenticated through modern BAX upregulation and intrinsic apoptotic signalling. Additionally, *R. coriaria* (Alsamri et al., 2021bib_alsamri_et_al_2021) is a tissue-fortifying astringent; *V. indica* (Venkateshwarlu et al., 2011) resin treats ulcers; *S. officinalis* (Chandra et al., 2021) is a depurative; *A. lebbeck* (El-Sayed et al., 2026) is anti-toxic; and *C. melo* (Asif et al., 2014) variants are used for cooling and detoxification. Modern nanotechnology validates these claims, utilizing complex natural capping layers synthetic counterparts lack. Crucially, however, their definitive impact on improving clinical parameters such as tumour targeting and reducing systemic toxicity remains preliminary and demands rigorous, comparative *in vivo* validation to be conclusively established.

## 3 Discussion

Integrating ethnopharmacological nanotechnology into breast oncology is a transformative clinical frontier. Phytogenic zinc oxide nanoparticles, synthesized from sources like *Z. officinale* and *R. coriaria*, demonstrate potent anticancer efficacy. Beyond reactive oxygen species (ROS) (Checa and Aran, 2020), these biogenic structures drive programmed cell death via mitochondrial dysfunction, BAX upregulation, cell-cycle arrest, and microRNA modulation (Lee et al., 2020).

This pro-oxidant behaviour coexists with the intrinsic antioxidant properties of the phytochemical capping agents through a compartmentalized, biphasic mechanism. Externally, the antioxidant cap stabilizes the nanostructure and scavenging peripheral radicals, maintaining systemic biocompatibility. However, upon cellular internalization, the nanoparticle acts as a “Trojan Horse”; the acidic lysosomal microenvironment triggers rapid dissolution of the ZnO core into a toxic influx of free Zn^2+^ ions. This sudden intracellular ionic surge directly disrupts the mitochondrial electron transport chain, shifting the biochemical balance to drive a catastrophic, fatal surge of intracellular ROS that completely overwhelms the localized antioxidant capacity of the host cell.

Beyond classical apoptosis, ZnO nanoparticles concurrently activate ferroptosis (Chen et al., 2022; Wang et al., 2021). This introduces significant complexity: oxidative stress and rapid zinc ion dissolution trigger mitochondrial collapse while depleting glutathione and initiating iron-dependent lipid peroxidation. This dual activation suggests plant-capped formulations overwhelm malignant cells through cross-talking lethal pathways. Acknowledging this ferroptotic potential is vital to bypass apoptosis-resistant phenotypes. Furthermore, transitioning to 3D spheroids accurately replicates physiological tumour barriers, yielding benchmarks like an IC50 of 14.7 μg/mL and total destruction using ginger-mediated formulations.

Phytogenic ZnO NPs offer superior biocompatibility over chemically synthesized counterparts, which often rely on hazardous reagents and leave toxic residues. By using natural metabolites like gingerols or withanolides, phytogenic synthesis creates nanostructures that integrate anti-inflammatory, antioxidant, and antiproliferative synergies, providing a distinct advantage in targeted anticancer therapeutic indices.

Furthermore, in specific cell-line assays, biogenic structures demonstrate favourable cellular selectivity profiles (e.g., against HUVEC cells), though this represents a glaring evidentiary gap across the broader field. The reliance on a single study utilizing a non-cancerous comparator is insufficient to justify broad claims of selective toxicity. Moving forward, the inclusion of normal mammary epithelial controls, such as MCF-10A, must be adopted as a mandatory minimum standard to definitively establish off-target safety thresholds for clinical translation in breast oncology.

This comprehensive therapeutic integration directly underscores the core novelty of this work when positioned against generalized literature, such as the 2024 review by Rodríguez-Barajas et al. on breast and prostate ZnO interactions (Rodríguez-Barajas et al., 2024). Rather than simply cataloguing metal-extract combinations, this review differentiates itself by critically evaluating the deconstruction of multi-layered architectural tissue barriers alongside precise epigenetic, cell-cycle, and apoptotic regulatory mechanisms.

Optimizing these nanomedicines requires deconvoluting the contributions of intact nanoparticles, dissolved Zn^2+^ ions, and metabolite capping agents. Therapeutic activity is tripartite: intact structures drive ROS production, lysosomal dissolution releases Zn^2+^ to cause mitochondrial collapse, and metabolite caps provide epigenetic synergies. To isolate these mechanisms, studies must utilize ultrafiltration or equilibrium dialysis to separate leached ions from nanoparticles. Furthermore, applying the chelator TPEN can neutralize ionic toxicity, while comparative arms with bare chemical ZnO and pure extracts will establish accurate, distinct pharmacokinetic models.

Preclinical barriers restrict clinical translation. Botanical extracts require rigorous metabolomic standardization and bio-guided fractionation to ensure reproducibility. Furthermore, a lack of deep *in vivo* validation, ADME profiling, and multi-organ toxicity metrics leaves systemic stability and clearance unresolved. Addressing these gaps, specifically long-term safety, metabolic fate, and biodistribution, is essential. Future pipelines must prioritize standardizing biophysical surface interactions, such as protein corona formations, alongside scalable green fabrication, before assessing first-in-human toxicological parameters.

## 4 Limitations

Despite the diverse mechanisms of action detailed above, ranging from ROS induction and caspase activation to S-phase arrest and microRNA modulation, a unifying critique of the field reveals significant methodological constraints. The primary constraint of current research into phytogenic ZnO NPs is the pervasive reliance on 2D monolayer cultures, which fail to duplicate the dense architectural barriers, complex extracellular matrices, and interstitial pressures, and hypoxia gradients inherent to solid breast tumours.

Static models fail to predict nanoparticle penetration or efficacy. The widespread absence of multi-cellular tumour spheroids (MCTS) or patient-derived xenografts (PDX) limits understanding of interactions within the dynamic tumour microenvironment, including stromal barriers and immune infiltrates. Furthermore, the lack of comprehensive *in vivo* validation and toxicity data against non-malignant lines, with only one study utilizing a HUVEC comparator, prevents establishing a selective therapeutic index. The field urgently requires comparative data against normal mammary cells, such as MCF-10A, to validate these selectivity claims and bridge the translational gap.

Systematically, while approaches like microRNA modulation are innovative, the underlying complexity of gene regulatory networks poses risks of undiscovered off target effects. Moreover, the inherent variability of biogenic capping agents introduces significant challenges in the standardization of nanoparticle size and morphology, which complicates batch-to-batch reproducibility. Because the native capping involves complex, unpurified matrices of secondary metabolites, systematically isolating the specific active phytoconstituents responsible for driving therapeutic synergy or maintaining colloidal stability remains a formidable analytical challenge. Additionally, the reliance on larger biogenic clusters, such as the 171–195 nm ginger-mediated formulations, introduces critical systemic liabilities. Outside controlled cell cultures, dimensions exceeding 150 nm drastically alter *in vivo* biodistribution by accelerating hepatic and splenic macrophage phagocytosis via the reticuloendothelial system (RES), risking premature peripheral clearance before tumour accumulation can occur.

Critical gaps remain, particularly the lack of comprehensive ADME profiling (Obuotor et al., 2023), *in vivo* pharmacokinetics, and biodistribution, leaving systemic stability and chronic toxicity unknown. Without longitudinal studies, risks like heavy metal accumulation and inflammatory cascades remain unquantified. Furthermore, the literature lacks data on GMP manufacturability, surface corona reproducibility, and human-equivalent dosing, making safety margins speculative. Additionally, botanical variability complicates batch-to-batch consistency, while microRNA modulation poses risks of off-target epigenetic effects. Exhaustive preclinical validation and rigorous toxicological mapping are essential to overcome these hurdles and establish reliable clinical safety.

Moreover, a significant compositional gap in the current literature is the frequent omission of botanical voucher specimen numbers (except Umar et al., 2019 and D’Souza et al., 2021) and the failure to explicitly validate plant nomenclature against recognized taxonomic databases such as Kew’s Plants of the World Online (POWO). Without these fundamental tracking details, reproducing the exact ethnopharmacological extracts remains impossible. While the cytotoxicity of biogenic nanoparticles is conventionally reported in mass/volume units (µg/mL), the lack of molar concentration reporting for the associated free Zn^2+^ ions or isolated bioactive phytochemicals limits strict pharmacological reproducibility.

## 5 Future scope

To bridge the clinical gap, future research must utilize 3D spheroids and murine models to confirm physiological barrier penetration. Benchmarking against clinical standards like Tamoxifen is vital for quantifying efficacy. Studies should integrate multi-targeted evaluations, incorporating S-phase arrest and ROS-induced mitochondrial dysfunction, to enhance *S. officinalis* L. (Caryophyllaceae) ZnO-NP potency. Furthermore, implementing rigorous ADME profiling and standardizing non-malignant comparators like MCF-10A is essential to ensure systemic clearance and long-term safety.

Crucially, establishing a rigorous cascade of intermediate translational milestones is vital to optimize biological dosing, evaluate systemic safety, and validate preclinical therapeutic indexes. This pathway requires four interconnected phases: investigating nanoparticle–protein corona dynamics to understand how adsorbed biomolecules dictate *in vivo* targeting and clearance; establishing Good Manufacturing Practice (GMP) scaling protocols to standardize the reproducibility of plant capping layers despite botanical variability; executing Investigational New Drug (IND)-enabling toxicology studies across multi-species and genotoxicity assays to map organ-specific safety thresholds; and setting precise parameters for Phase I dose-finding assays in living systems to determine the Maximum Tolerated Dose (MTD) and human safety margins.

To reach clinical trials, research must transition to microfluidic organ-on-a-chip platforms for evaluating kinetic clearance. Integrating liquid chromatography-mass spectrometry with bio-guided fractionation is crucial to identify metabolite capping. Toxicological assessments must expand to transcriptomic and proteomic profiling on healthy tissues to monitor off-target effects. Finally, standardized protocols are needed to decouple cytotoxicity of zinc ions from nanoparticle and phytochemical effects. Future studies must report precise molar units for decoupled components, aligning botanical nanomedicine with ethnopharmacological best practices.

Reconciling ethnopharmacological antioxidant properties with ZnO-mediated oxidative stress will establish an evidence-based framework for clinical oncology translation. Future frameworks must explicitly model this antioxidant/pro-oxidant interface by using advanced cell-free and intracellular radical tracking assays.

Quantifying the precise thermodynamic thresholds where plant-derived capping layers transition from radical-scavenging shields into pro-oxidant executioners inside malignant vacuoles will be essential to establish predictable safety profiles and reproducible clinical outcomes.

## 6 Conclusion

The ethnopharmacological synthesis of ZnO NPs represents a transformative frontier in breast cancer therapy, merging sustainable nanotechnology with potent bioactive metabolites. These biogenic platforms have demonstrated significant potential in inducing apoptosis via ROS-mediated mitochondrial dysfunction, BAX upregulation, and precise cell cycle arrest. Notable benchmarks, such as tumour suppression comparable to Tamoxifen in select *in vivo* models, underscore their therapeutic viability. However, for these nanomedicines to move into clinical oncology, the field must transcend simplified laboratory models. Ultimately, constructing a realistic translational pathway demands a systematic progression from translational cell culture to rigorous preclinical validation. Prioritizing accurate biodistribution modelling, exhaustive long-term toxicity evaluations and detailed metabolic clearance studies is essential to establish the foundational safety and therapeutic efficacy required for modern oncological practice. By integrating 3D architectural modelling with rigorous systemic validation and exhaustive pharmacokinetic profiling, these eco-friendly nanostructures can be validated as high-potency, multi-targeted interventions.

## Footnotes

1
https://www.healthdata.org/news-events/newsroom/news-releases/lancet-oncology-breast-cancer-remains-most-common-cancer-among
